# Surgery

**Published:** 1878-05

**Authors:** 


### Surgery.

Sudden Death From Severe Burns.—Ponfick. (*Berl. Klin. Wochenschr*, 1877, No. 46.)

P. has studied with F. Schmidt the effects of burns on dogs. Within a few minutes he found the red corpuscles of the blood dividing into an immense number of minute colored particles. These disappeared within a short time, thereby producing changes in the spleen, osseous marrow and kidneys. By the latter organs, the hemoglobine thus liberated in the blood is removed, not, however, without causing inflammation of the parenchyma. Since this sudden extensive destruction of red corpuscles seems to be an important factor in the grave results of burns, Ponfick suggests transfusion as a therapeutic experiment.

Dislocation of the Spine.—Dr. T. E. Little, of Dublin Pathological Society. (Rep. in *Dublin Jour. of Med. Sci.*, March, 1878.)

A case of genuine and uncomplicated dislocation of the spine was presented, almost a surgical anomaly.

A lad aged 18, fell, striking on the back of the head and shoulders, on a coal heap. Stunned for the moment, he was able to walk into the hospital ward from the cab. When Dr. L. first saw him, there was complete loss of power of both upper extremities, with diminution of sensibility; muscular power of lower limbs slightly impaired, head somewhat retracted but not rigid, intense pain upon effort to move the head, dejections voluntary, temp. 99½°. No displacement of vertebræ could be detected, even by examination through the pharynx. There was flushing of the surface of the upper parts of the body; a symptom Dr. L. had noticed in other cases of spinal injury. Within the four days following the tenth, the patient developed a well marked case of "Cruveilhier's Paralysis," muscular atrophy of the forearms being complete. There was also a peculiar glossy appearance of the hands (such as has been alluded to by Weir Mitchell).

Subsequently he had symptoms of Binz's "Paralytic fever,"

checked by antiperiodic doses of quinine; and he eventually emaciated, with contractures of lower extremities, but without atrophy. Retention of urine and diaphragmatic respiration, owing to atrophy of the *intercostales*, were accompanying features of the case. He succumbed at last to a gradually increasing apnœa, dying on the eighty-sixth day.

Autopsy revealed symmetrical dislocation forwards of the fifth cervical vertebra upon the sixth, displacement of the articular processes being complete. The anterior and posterior common ligaments were both intact. The spinal canal was narrowed to half its caliber, the point of greatest constriction being—as usual—the exact site of displacement. No microscopical examination of the cord was made. The muscles of the upper extremity were reduced to mere sheaves of connective tissue, with rows of fat globules, and here and there a muscle fiber apparently healthy.

Incised Penetrating Wounds of Knee Joint—Dr. A. D. Stevens, (*Canada Medical Record*, *March*, 1878.)

The first case occurred in February, 1873, and was in the person of a boy about sixteen years of age, of healthy appearance himself, as well as his parents. He had been preparing wood for sugar making, and accidently struck his knee with the axe, inflicting a wound about an inch in length upon the upper and outer border of the patella, and exposing the joint to that extent. He did not stop using the injured limb until an active inflammation set in, when my services were asked. Upon visting him, I found all the symptoms of inflammation well marked, and the limb placed in the usual position, with a view of lessening pain. I at once gave him alterative doses of grey powders with Dover, and cold applications were placed upon the affected joint. After the more acute symptoms had subsided, I gave him iodide of potassium, with compound tincture gentian, and painted the knee with tincture of iodine. At this time, I was also able to place a well adjusted splint upon the posterior portion of the limb, in such a manner as to secure perfect rest of the joint, with the limb straightened. But few days passed before the presence of pus in the cavity of the joint was evident, but as the opening made by the axe still existed, I did not interfere. I turned the boy over upon his belly, when a large amount of pus escaped from the opening.

The remainder of the treatment consisted principally in keeping the limb in the position forced by the splint, and doing for him whatever constitutionally he might require. The joint filled at least half a dozen times with pus, but was as often emptied by turning him over. The patient was kept in bed with the splint securely fastened to the leg till all appearances of disease had left, when he was allowed to use it cautiously. He has to-day as valuable a limb as he ever had.

The second case occurred in the month of August, 1875, and was in the person of a boy about fourteen years old. Like the preceding case he was healthy, and of healthy origin. The cut was made with the axe, as in the former instance, but nearly opposite the site of the other and about the same length. I did not see this boy, however, till suppuration had taken place, so that he had only to be turned over to relieve the joint of its contents. It only filled once, fortunately, and, with the aid of the splint already noticed, and alterative doses of iodide of potassium combined with a bitter tonic, and free painting of the joint, soon all traces of disease disappeared. About the middle of the following October he was able to do full duty upon the farm.

With reference to the third case I promised to speak about, I might say that, like the other two, he was apparently healthy and of robust parentage, while his age was about thirty years. He is married, and his occupation that of carpenter. While working at the frame of a building, his adze, from some cause or other, missed and struck him a blow just underneath, and a little to the right of the patella, causing a wound fully an inch in length, and penetrating the joint. I saw him within two hours of the accident. He had lost only a trifling amount of blood, but the wound was gaping to such an extent that the synovial membrane was visible for more than the length of the cut. Thus you will perceive the cavity of the joint received all the fresh air you could ask for. This fellow I strapped with the ordinary adhesive straps in such a manner as to prevent any motion whatever of the joint, and enjoined him on no account to step upon the foot. He returned twice afterwards, for a renewal of the dressing, which, with a simple wash of carbolic acid, was all that was required for the cure of the wound. The wound healed by the first intention, and consequently no inflammation supervened, or, in fact, any other untoward event. In less than four weeks he was as well able to work as ever.

These are all the leading facts and particulars of the three cases, with the exception of the passive motion used in order to prevent anchylosis, and which I forgot to mention in the proper place.

